# Should Preachers Pay

**Published:** 1893-03

**Authors:** 


SHOULD PREACHERS PAY.—The writer received from a well known specialist a letter worded as follows: "Dear Doctor, this will introduce the Rev. ——. He is under my care for —— and asked me to recommend a good dentist and I have taken the liberty of sending him to you. Mr. —— will not be able to pay you a fee but will no doubt be able to send you some patients. Yours very truly, ——.

It is the opinion of many dentists and physicians that the custom of not charging any fee for professional services rendered to a minister of the Gospel or his family does its greatest harm to the clergy, and that it is with them that the reform ought to originate. Under the existing custom if a preacher and his family are being attended by a doctor in whom they lose confidence, it must be very awkward to demand that the doctor cease his gratuitous visits. It is not every one who is blessed with a person, like the maid described, who gave the doctor a hint. A lady had been ill and under medical treatment for a long time. As she grew no better all the while, she became distrustful of her physician's skill and did not wish to see him, and yet was not bold enough to tell him so. She communicated her state of mind to her maid.

"Lave' 'im to me, mum, lave' 'im to me!" said the girl.

By-an by the doctor came to the door, and Bridget opened it about an inch.

"Sorry, sir," said she, but ye can't come in to-day, docthor!"

"Can't come in? How's that?"

"The misthress do be too ill for to see ye the day, sir."

We do not believe that professional services can never be conscientiously dispensed except on a commercial basis, otherwise medicine or dentistry is only a trade, after all, and its dignity is gone. The custom should be abolished in the interest of both the clergyman and the doctor, among other reasons, because the clergyman is put at a disadvantage since he cannot always have his choice of attendance but must take his physician or dentist out of his congregation, if he is to be found there. Moreover since he makes no payments in money, he must feel a delicacy in calling for service to the extent which he would desire. Personally the writer has paid his family physician his fees, free of deductions, and has collected from him regular mimimum office charges for dental services. On the other hand he has never sent any bill for services rendered the "pastor" but has charged members of the family. It is not known that any rule prevails among dentists.

